# Suppression of NK cell-mediated immunosurveillance by IL-35 drives tumor progression in EGFR-mutant non-small cell lung cancer

**DOI:** 10.3389/fonc.2025.1692801

**Published:** 2025-11-20

**Authors:** Na Li, YinSong Zhang, XiaoJie Zhang, HaiZhi Wang, Fang Yang, FengJu Zhou, DaQing Wang, Yan Zhang

**Affiliations:** 1 Department of Oncology, Hebei Medical University, Shijiazhuang, Hebei, China; 2 Department of Medical Oncology, Hengshui People’s Hospital, Hengshui, Hebei, China; 3 Department of Neurosurgery, Hengshui People’s Hospital, Hengshui, Hebei, China; 4 Department of Interventional Therapy, Hengshui People’s Hospital, Hengshui, Hebei, China; 5 Department of Laboratory Medicine, Hengshui People’s Hospital, Hengshui, Hebei, China; 6 Department of Medical Oncology, Shijiazhuang People’s Hospital, Shijiazhuang, Hebei, China

**Keywords:** IL-35, EGFR mutation, NSCLC, NK cells, immune suppression, cytokines

## Abstract

**Background:**

Interleukin-35 (IL-35) is an added member of the IL-12 heterodimeric cytokine family, composed of two subunits: EBI3 and P35 subunits, implicated in tumor immune evasion. This study investigates the expression and immunosuppressive role of IL-35 in epidermal growth factor receptor (EGFR)-mutant non-small cell lung carcinoma (NSCLC), with a focus on its interaction with natural killer (NK) cells.

**Methods:**

Eighty-two NSCLC tissue samples (47 EGFR-mutant and 35 wild-type) were assessed for IL-35 expression via immunohistochemistry (IHC) targeting EBI3 and P35. ELISA, Western blot, and PCR validated protein and mRNA expression in fresh tissues (n = 14). The degree of NK-cell infiltration was evaluated as the percentage of NKp46-positive cells among CD45-positive cells. Peripheral NK cells were isolated from healthy donors and subjected to IL-35 treatment. Functional assays included CCK8, flow cytometry for CD3-CD56+ cells and NKG2D, ELISA for cytokine secretion, and cytotoxicity assays on NSCLC cell lines. *In vivo*, H1975 and PC-9 xenograft models with EGFR-sensitive mutations were used to assess the effects of IL-35 on tumor growth and NK-cell infiltration.

**Results:**

IL-35 was significantly overexpressed in EGFR-mutant NSCLC tissues, with strong concordance between EBI3 and P35(r = 0.795, P < 0.0001). High IL-35 expressions associated with larger tumor size (χ2 = 16.140, P = 0.000) and EGFR mutation status (χ2 = 4.843, P = 0.028). IL-35 expression levels were associated with patient prognosis in both the overall population and the EGFR-mutant subgroup (Kaplan–Meier, P < 0.05).IL-35 expression inversely correlated with NKp46- cell density (r = -0.526, P = 0.000). The percentage of NKp46-positive cells among CD45-positive cells differed significantly between mutant and wild-type NSCLC tissues (t=-9.083,P=0.000). IL-35 inhibited NK cell proliferation and function *in vitro*, reducing CD3-CD56+ cell proportion (F = 101.3, P < 0.0001), NKG2D expression (F = 49.29, P = 0.0002), and cytokine secretion (IFN-γ, F = 252.388, P = 0.000, Perforin, F = 39.372, P = 0.000, Granzyme, F = 1001.822, P = 0.000); Conditioned medium from IL-35-treated NK cells enhanced proliferation, invasion, and migration of NSCLC cell lines. *In vivo*, IL-35 promoted tumor growth, while IL-35 neutralization reduced tumor size. The proportion of NKp46^+^ cells among CD45^+^ cells differed significantly across the control, IL-35, and IL-35 neutralizing antibody groups (One-way ANOVA, P = 0.000). The *in vivo* results in mice indicated that IL-35 expression remained inversely related to NKP46 expression (r = -0.753, P = 0.000).

**Conclusion:**

IL-35 is upregulated in EGFR-mutant NSCLC and mediates immune suppression by impairing NK cell activity. Targeting IL-35 may offer a therapeutic avenue to restore NK cell function and enhance anti-tumor immunity.

## 1 Introduction

Lung carcinoma is responsible for the majority of cancer-related deaths on a global scale, and its subtype non-small cell lung carcinoma (NSCLC) comprises nearly 85% of all lung cancer diagnoses (1). Of Asian populations, epidermal growth factor receptor (EGFR) mutations are particularly prevalent in NSCLC patients, accounting for a mutation rate of 30-50% (2). While EGFR tyrosine kinase inhibitors (TKIs) have fundamentally changed therapeutic outcomes, inevitable development of acquired resistance remains a significant clinical challenge. Recent studies have revealed that EGFR mutations can profoundly alter the tumor immune microenvironment, promoting immunosuppressive cells including myeloid-derived suppressor cells, an increased expression of regulatory T cells, and alterations in cytokine profiles (3, 4). However, the precise mechanisms underlying EGFR mutation-mediated immunosuppression remain incompletely understood.

Interleukin-35 (IL-35), a cytokine that belongs to the IL-12 family, has emerged as a potent immunosuppression factor prevailingly produced by regulatory T cells and other immunosuppressive cell populations (5). In various malignancies, including pancreatic and colorectal cancers, IL-35 has been shown to facilitate tumor development via suppressing antitumor immune responses (6). Given that EGFR mutations are associated with increased infiltration of immunosuppressive cells, it is plausible that IL-35 expression may be elevated in EGFR-mutant NSCLC tumors. However, whether EGFR mutations directly influence IL-35 production in the tumor microenvironment remains unexplored.

As essential elements within the innate immune system, natural killer (NK) cells significantly contribute to tumor surveillance and elimination through direct cytotoxicity and cytokine production (7). In lung cancer, high NK infiltration or more functional NK phenotypes have been correlated with better prognosis and improved responses to immunotherapy. However, within solid tumors, NK cells often become dysfunctional or excluded, owing to a hostile microenvironment including hypoxia, lactic acidosis, TGF-β, metabolic stress, and suppressive factors (e.g. regulatory cytokines) (8). IL-35 was further shown to drive NK toward an ILC1-like phenotypic conversion in a TGF-β–dependent manner (autocrine loop) (9). In addition, *in vivo*, B cell–derived IL-35 (via a STING–IL-35 axis) was shown to reduce NK proliferation and blunt NK-driven antitumor immunity in murine tumor models (10).While IL-35 has been implicated in suppressing various immune cell functions, its specific impact on NK cell activity in the context of EGFR-mutant NSCLC remains largely unknown. Understanding this interaction may provide novel perception of the immune suppressive mechanisms underpinning EGFR-driven tumorigenesis and identify promising therapeutic targets to overcome resistance.

## 2 Materials and methods

### 2.1 Immunohistochemistry

Upon approval by the Ethics Committee of Hengshui People’s Hospital (Hebei, 053000 China), a total of 82 samples obtained from NSCLC patients by surgical resection or needle biopsy were included in the analysis. Immunohistochemical (IHC) staining was performed for EBI3(Abcam), p35(Abcam), NKP46 (Affinity Biosciences)andCD45(Abcam),according to the manufacturers’ protocols. The intensity of staining (IS) was scored on a scale ranging from 0 to 3, with 0 indicating negative staining, 1 weak, 2 moderate and 3 strong. The extent of staining was quantified as percent of cells stained positive: 0 (0%), 1 (1–25%), 2 (26–50%), and 3 (51–100%). The final IHC score was determined as the sum of these two scores, yielding a range of 0 to 6. The results were categorized into four levels: negative (0), low (1-2), moderate (3-4), and high (5-6). Through the statistics analysis, expression levels were further dichotomized into low (scores 0-2) and high (scores 3-6) groups. The percentage of NKp46-positive cells among CD45-positive cells was calculated as the mean value obtained from five randomly selected fields at 20× magnification. Two independent pathologists, blinded to the clinical data, evaluated all slides to ensure objectivity and reproducibility. Discrepancies were resolved through consensus review.

### 2.2 Enzyme-linked immunosorbent assay

Fresh tumor tissues were subjected to homogenization in PBS with protease inhibitors and then to centrifugation at 12,000 × g for 10 min. Following centrifugation, supernatants were gathered and analyzed by performing an IL-35 ELISA with an R&D Systems kit as per manufacturer’s instructions. NK cell supernatants, collected after 24-hour culture, were tested for IFN-γ (Thermo Fisher), perforin (Thermo Fisher, BMS2306), and granzyme B (Thermo Fisher) by ELISA. Absorbance was determined at a wavelength of 450 nm using a Synergy HTX microplate reader (BioTek).

### 2.3 Western blotting technique

Tumor tissues were prepared in RIPA lysis buffer (Solarbio, R0010) containing protease and phosphatase inhibitors to extract proteins. A bicinchoninic acid (BCA) assay kit (Servicebio) was used for the quantification of protein concentration. Then 30 µg of protein (equal amounts) were transferred onto PVDF membranes (Millipore) after being resolved on SDS-PAGE. Following blocking with 5% non-fat milk, the membranes were incubated with primary antibodies against EBI3 (1:1000, Abcam), β-actin (1:5000, Affinity Biosciences) and P35 (1:1000, Abcam) for an entire night at 4 °C. The next step was to incubate at room temperature for 1 hr with HRP-conjugated secondary antibodies. An ECL substrate kit (ShareBio) was employed to detect signals and a ChemiDoc system (Bio-Rad) was subsequently performed to visualize them.

### 2.4 Real-time PCR or qPCR

TRIzol Reagent was purchased from Invitrogen™ to isolate total RNA from NSCLC tumor tissues. A One microgram of total RNA was reverse-transcribed using the PrimeScript RT Reagent Kit (GenStar). SYBR Green Master Mix from GenStar was conducted on a StepOnePlus Real-Time PCR System to do the qPCR trail. Primer sequences were:EBI3: Fwd 5′-CCTTCATTGCCACGTACAGGCT-3′, Rev 5′-CGGTGACATTGAGCACGTAG -3′;P35 (IL-12A): Fwd 5′-AGTTGCCTGGCCTCCAGAAA -3′, Rev 5′-CTCCACCTGGTACATCTTCA -3′;β-actin: Fwd 5′-CACGATGGAGGGGCCGGACTCATC -3′, Rev 5′-TAAAGACCTCTATGCCAACACAGT. -3′Relative gene expression data obtained from qPCR were analyzed via the 2^−ΔΔCt method.

### 2.5 PBMC isolation and NK cell expansion

Heparinized tubes were used to draw peripheral blood from healthy donors, and peripheral blood mononuclear cells (PBMCs) were separated peripheral blood utilizing Ficoll-Paque PLUS (GE Healthcare), a ready-to-use aqueous medium for density gradient centrifugation. The NK cell expansion kit used in this study was obtained from STEMERY (Catalog No.: CT-00130). NK cells were cultured in RPMI-1640 medium provided by Gibco and enriched with 10% FBS, IL-2 (1000 IU/mL), and IL-35 (100 ng/mL(10), Cohesion Biosciences,CRP2755). NK cell purity was evaluated using flow cytometry based on CD3^−^CD56^+^ expression.

### 2.6 Flow cytometry

Cells were labeling using monoclonal antibodies conjugated to a fluorochrome: anti-CD3-APC (Cohesion Biosciences), anti-CD56-PE (Cohesion Biosciences), and anti-NKG2D-FTIC (Biolegend). After being incubated for half an hour at 4 °C away from light, cells were rinsed and assessed with a FACSCanto II (BD Biosciences). The findings were processed using FlowJo software (Tree Star, Inc.). The proportion of CD3^−^CD56^+^ NK cells and NKG2D expression were quantified.

### 2.7 Cell functional assays

CCK-8 Assay: NSCLC cells were placed in a 96-well plate at 5×10³/well and cultured for 0, 6, 12, 24 and 36 hours. Cell growth was evaluated using the CCK-8 kit (ZETA), and the absorbance was recorded at 450 nm. Transwell Invasion Assay: 24-well Transwell inserts (Thermo Fisher) coated with Matrigel (BD) were used. Cells (1×10^5^) were plated in the upper chamber, suspended in a cell culture medium that does not contain animal-derived serum; the addition of 10% FBS to the lower chamber served as a chemoattractant. Under a microscope, invading cells went into five randomized fields after fixation and staining with 0.1% crystal violet for a whole day. Wound Healing Assay: In 6-well plates, cells were cultured to 90% confluence before being scraped with the tip of a sterile pipette. Washing was followed by serum-free cell culture. Images were captured at 0h, 6h, 12h and 24h under an inverted microscope. Migration was estimated by measuring the scratch area.

### 2.8 *In vivo* xenograft tumor model

Beijing Vital River Laboratory Animal Technologies Co., Ltd. supplied the female BALB/c nude mice, which were 4–6 weeks old and of SPF grade, and were kept in a pathogen-free environment. Suspensions of 5 × 10^6^; H1975 or PC-9 cells in 100 μL PBS were implanted subcutaneously into the right axilla of each mouse. Starting on day 7 post-inoculation, length and width of the tumor were calculated every 3days by a caliper so as to figure out tumor volume as (length × width²)/2. Mice were randomized into three groups (n = 3/group): control group, IL-35-treatment group (Cohesion Biosciences), and IL-35-neutralizing antibody group (Sigma-Aldrich).Mice in the control group received 200μL PBS via intraperitoneal injection (i.p.) every other day. The IL-35-treatment group was administered recombinant IL-35 protein at a dose of 0.75μg/mouse (i.p., every other day) (11). The group of neutralization with IL-35-specific antibodies received anti-EBI3 monoclonal antibody (clone V1.4C4.22, MABF848, Sigma-Aldrich) at an initial dose of 100μg/mouse via intraperitoneal injection and then at a 50 μg/kg maintenance dosage every other day (6).Tumor size was monitored every 3days after treatment initiation. On the 28th day after tumor formation (the 28th day of drug administration), the mice were euthanized by cervical dislocation, and the tumors were excised and weighed.

### 2.9 Statistics reasoning

Each experiment in the present study was done a minimum of three times. IBM SPSS Statistics 21 was leveraged to make a statistics analysis. Intergroup differences were assessed by one-way ANOVA, chi-square test, or Fisher’s exact test. In Spearman’s rank correlation, its coefficient was a strong measure of association between two variables. Kaplan–Meier survival analysis was performed. When the p-value was smaller than 0.05, it was deemed to be statistically significant.

## 3 Results

### 3.1 Expression of IL-35 is elevated in EGFR-mutant NSCLC and correlates with tumor size and EGFR mutation status

**Figure 1:**
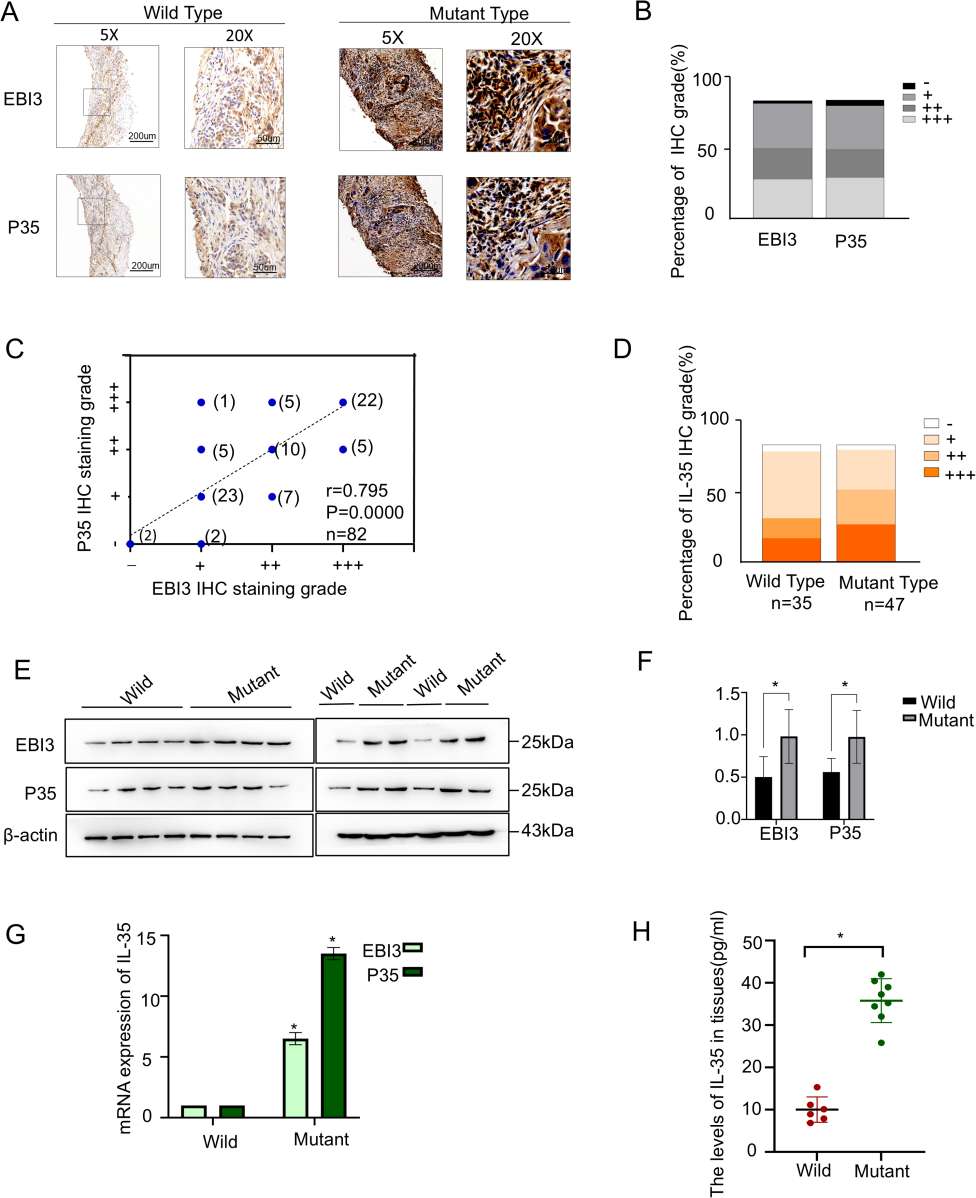
IL-35 is upregulated in EGFR-mutant NSCLC tissues. **(A)** Representative immunohistochemical staining of two IL-35 subunits (EBI3 and P35) in EGFR wild-type (left) and EGFR-mutant (right) NSCLC tissues. Scale bars: 50 μm.**(B)** Quantification of IL-35 subunit expression levels based on IHC scoring across the NSCLC cohort, shown as the proportion of samples with low, medium, and high expression.**(C)** Correlation analysis between EBI3 and P35 IHC expression scores in NSCLC tissue samples (n = 82, Spearman’s correlation).**(D)** The proportion of IL-35 expression levels between the EGFR-mutant and wild-type groups. **(E)** Western blotting of EBI3 and P35 protein levels in EGFR-mutant and EGFR wild-type NSCLC tissues. β-Actin was taken as a loading control. **(F)** A bar graph showing the grayscale values of the Western blot results obtained using ImageJ. **(G)** A qPCR analysis of EBI3 and P35 mRNA expression in EGFR-mutant and wild-type NSCLC tissues. Data are presented as relative expression normalized to β-Actin. **(H)** ELISA measurement of IL-35 protein levels in tissue lysates from EGFR-mutant and EGFR wild-type NSCLC patients. The symbol * indicates that the difference is statistically significant.

IL-35, a heterodimer consisting of EBI3 and p35 subunits, was examined at the tissue level in 82 NSCLC specimens, including 47 EGFR-mutant and 35 EGFR-wild-type cases. Immunohistochemical staining suggested both EBI3 and p35 were highly expressed (**Figure 1A**) and showed strong concordance in expression levels (Spearman r=0.879, P<0.0001) (**Figures 1B, C**). EGFR-mutant NSCLC tissues expressed IL-35 in significantly higher levels compared with wild-type (χ² = 4.843, P = 0.028) (**Table 1**, **Figure1D**). Higher IL-35 level was positively linked to increased tumor size (**Table 1**). In both the overall population and the EGFR-mutant population, there were statistically significant differences in survival between the high and low IL-35 expression groups (P < 0.05, **Supplementary Figure 3**), indicating that IL-35 expression affects patient prognosis. To validate these findings, Western blot (**Figures 1E, F**), RT-PCR (**Figure 1G**), and ELISA analyses (**Figure 1H**) were performed on fresh NSCLC tissues (n = 14; EGFR-wild-type, n = 6; EGFR-mutant, n = 8). All three assays consistently confirmed significantly higher IL-35 levels expressed in both mRNA and protein of EGFR-mutant tissues than wild-type (T-test, Western blot, P < 0.05; RT-PCR, P < 0.05; ELISA, P < 0.05).

**Table 1:** Clinical characteristics of the patients.

Clinical characteristics/Subgroups	Total	IL-35 expression		χ^2^	P-value
		High	Low		
Gender	82			1.445	0.229
Male	45	22	23		
Female	37	23	14		
Age	82			1.785	0.182
<65	22	4	18		
≥65	60	20	40		
Smoke	82			1.961	0.161
Yes	44	21	23		
No	38	24	14		
Pathological type	82			0.003	0.955
Adenocarcinoma	77	40	37		
Squamous Cell Carcinoma	5	2	3		
Differentiation	82			0.455	0.500
Good/moderate	52	30	22		
Poor	30	15	15		
Pathological TNM	82				
I	1	1	0	1.438	0.697
II	3	1	2		
III	5	3	2		
IV	73	40	33		
Nodal involvement	82				
N0	25	10	15	3.591	0.309
N1	18	12	6		
N2	23	14	9		
N3	16	9	7		
Tumor size(cm)	82			16.140	0.000^*^
<5	42	14	28		
≥5	40	31	9		
EGFR Mutation Status	82			4.843	0.028^*^
Mutant	47	29	18		
Wild	35	13	22		
Types of Mutations	47			1.174	0.556
19del	18	11	7		
L858R	26	17	9		
19del/L858R/T790M	3	1	2		

*P < 0.05, considered statistically significant.

### 3.2 IL-35 expression negatively correlates with infiltrating NK cells in NSCLC tissues

**Figure 2:**
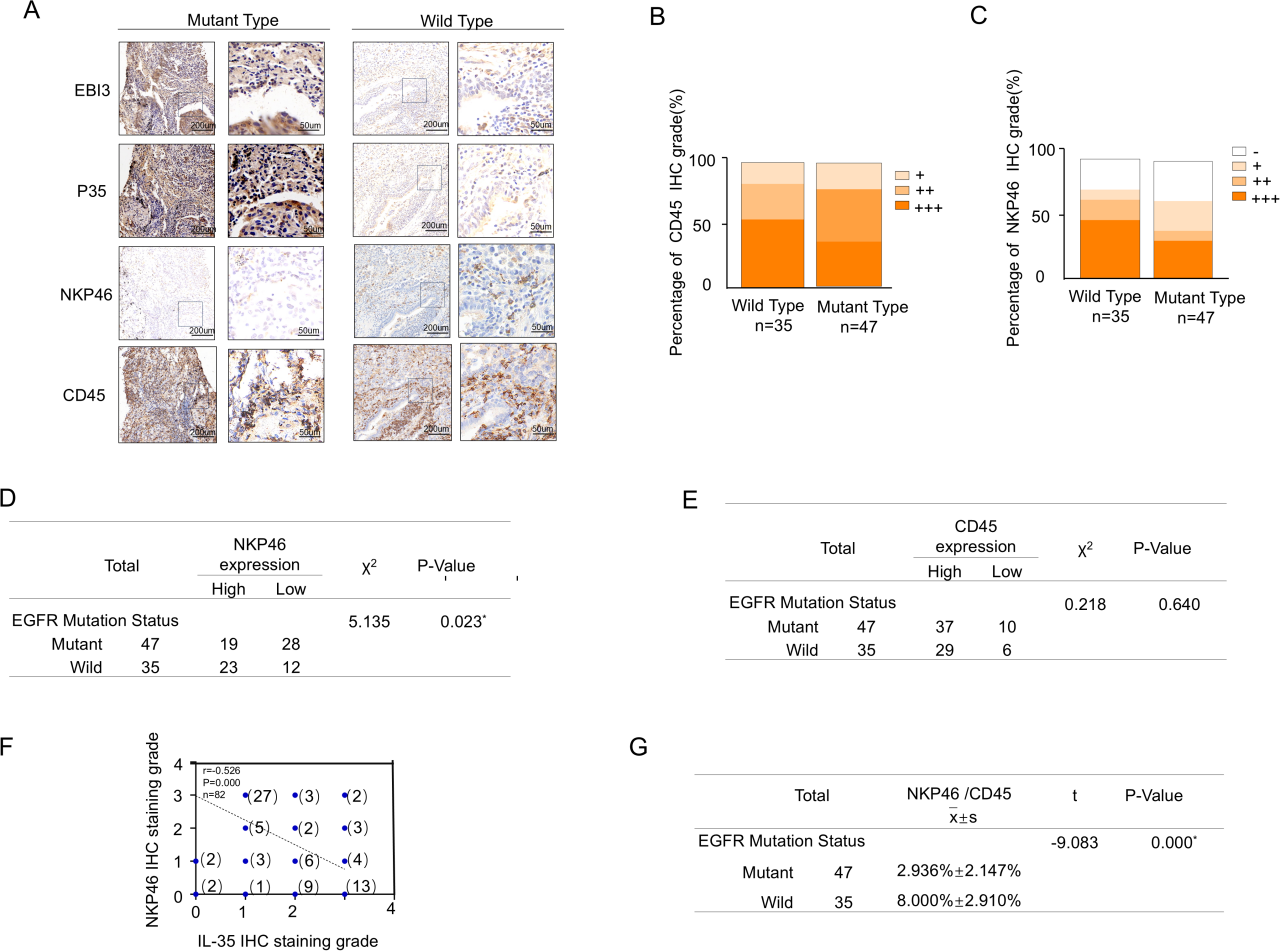
Correlation between NK and IL-35 cells in EGFR-mutated and EGFR-wild-type tissues. **(A)** Representative immunohistochemical staining images of NSCLC tissues with EGFR mutations (left panels) and wild-type EGFR (right panels), showing expression of the two IL-35 subunits (EBI3 and P35), the pan-leukocyte marker CD45, and the NK cell marker NKP46. **(B)** Proportion of tissue samples with high, moderate, and low CD45 expression in EGFR-mutant and EGFR-wild-type groups.**(C)** Proportion of tissue samples with high, moderate, and low NKP46 expression in EGFR-mutant and EGFR-wild-type groups. **(D)** Statistical comparison of NKP46expression levels between EGFR-mutant and EGFR-wild-type NSCLC tissues(χ² = 5.135, P = 0.023).**(E)** Statistical comparison of CD45 expression levels between EGFR-mutant and EGFR-wild-type NSCLC tissues ((χ² = 0.218, P = 0.640)). **(F)** Spearman correlation analysis demonstrating a significant inverse relationship between combined IL-35 (EBI3 + P35) expression and NKP46 expression levels in NSCLC tissues (r = –0.570, P < 0.001). **(G)** Statistical comparison of the NKP46^+^/CD45^+^ cell percentage between EGFR-mutant and EGFR-wild-type NSCLC tissues (t=-9.083,P=0.000).

Using NKp46/CD45 ratio as the indicator of NK cell infiltration, immunohistochemical staining was performed on the same 82 NSCLC tissue specimens. The mutant group exhibited higher IL-35 expression accompanied by reduced NKp46 expression, while CD45 expression showed no significant difference (**Figures 2A-C**). NKp46 expression showed a significant difference between EGFR-mutant and EGFR-wild-type tissues(χ² = 5.135, P = 0.023) (**Figure 2D**),whereas CD45 expression did not differ significantly (χ² = 0.218, P = 0.640) (**Figure 2E**). Moreover, IL-35 expression was negatively correlated with NKp46 expression (Spearman r = –0.526, P = 0.000) (**Figure 2F**), and the NKp46/CD45 ratio exhibited a statistically significant difference between the EGFR-mutant and EGFR-wild-type NSCLC groups (t=-9.083,P=0.000)(**Figure 2G**).

### 3.3 IL-35 suppresses NK cell proliferation and activation *in vitro*

**Figure 3:**
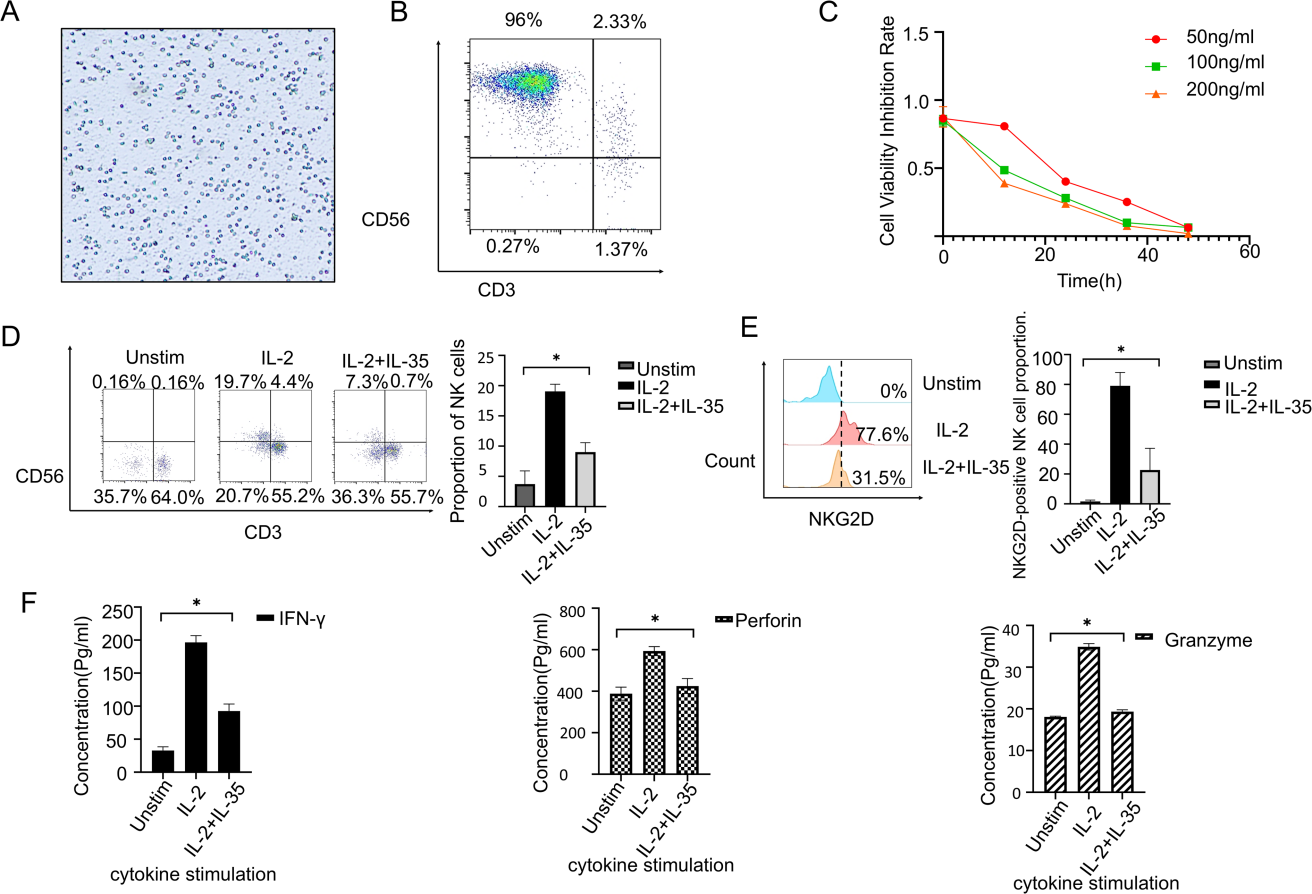
IL-35 suppresses the biological function of NK cells. **(A)** With the use of Ficoll-Hypaque density gradient centrifugation, PBMCs were extracted from healthy donors. **(B)** NK cells were expanded *in vitro* utilizing a commercial NK cell expansion kit, and the method for purity assessment was flow cytometry. The purity of CD3^−^CD56^+^ NK cells reached approximately 96%. **(C)** CCK-8 assay showed that IL-35 treatment significantly inhibited NK cell proliferation. **(D)** NK cells were categorized into three groups: unstimulated, IL-2-stimulated, and IL-2+IL-35-stimulated. After 24 hours of culture. Each group underwent flow cytometry to measure the proportion of CD3^−^CD56^+^ NK cells. **(E)** NKG2D expression in NK cells was investigated by flow cytometry after 24 hours of stimulation under the same conditions. **(F)** ELISA was performed to quantify IFN-γ, Granzyme B and Perforin secretion in the supernatants of NK cells cultured under the indicated conditions for 24 hours. (*) indicates statistically significant differences.

PBMCs were collected from healthy donors and utilized to expand NK cells *in vitro* (**Figure 3A**). Flow cytometry confirmed the purity of cultured NK cells at 98% (CD3^−^CD56^+^) (**Figure 3B**). A CCK-8 assay demonstrated a dose-dependent inhibition as well as time-dependent suppression of NK cell proliferation by IL-35 at concentrations of 50 ng/mL, 100 ng/mL, and 200 ng/mL(**Figure 3C**). Further, PBMCs were cultured for 24 hours under three conditions: unstimulated, IL-2-stimulated, and IL-2 + IL-35. According to flow cytometry, the percentage of CD3^−^CD56^+^ NK cells was 0.16%, 19.7%, and 7.3% (**Figure 3D**), respectively. IL-35 significantly inhibited IL-2-induced NK cell expansion (t-test,P < 0.05). Moreover, NKG2D expression on NK cells was drastically cut in the IL-2 + IL-35 group (31.5%) by comparison with the IL-2 group (77.6%, P < 0.05), while it was undetectable in unstimulated controls (**Figure 3E**). ELISA analysis of the culture supernatants revealed that IL-35 significantly suppressed IL-2-induced secretion of IFN-γ, perforin, and granzyme B by NK cells (t-test, P < 0.05) (**Figure 3F**).

### 3.4 IL-35 impairs NK cell-mediated anti-tumor effects on NSCLC cell lines

**Figure 4:**
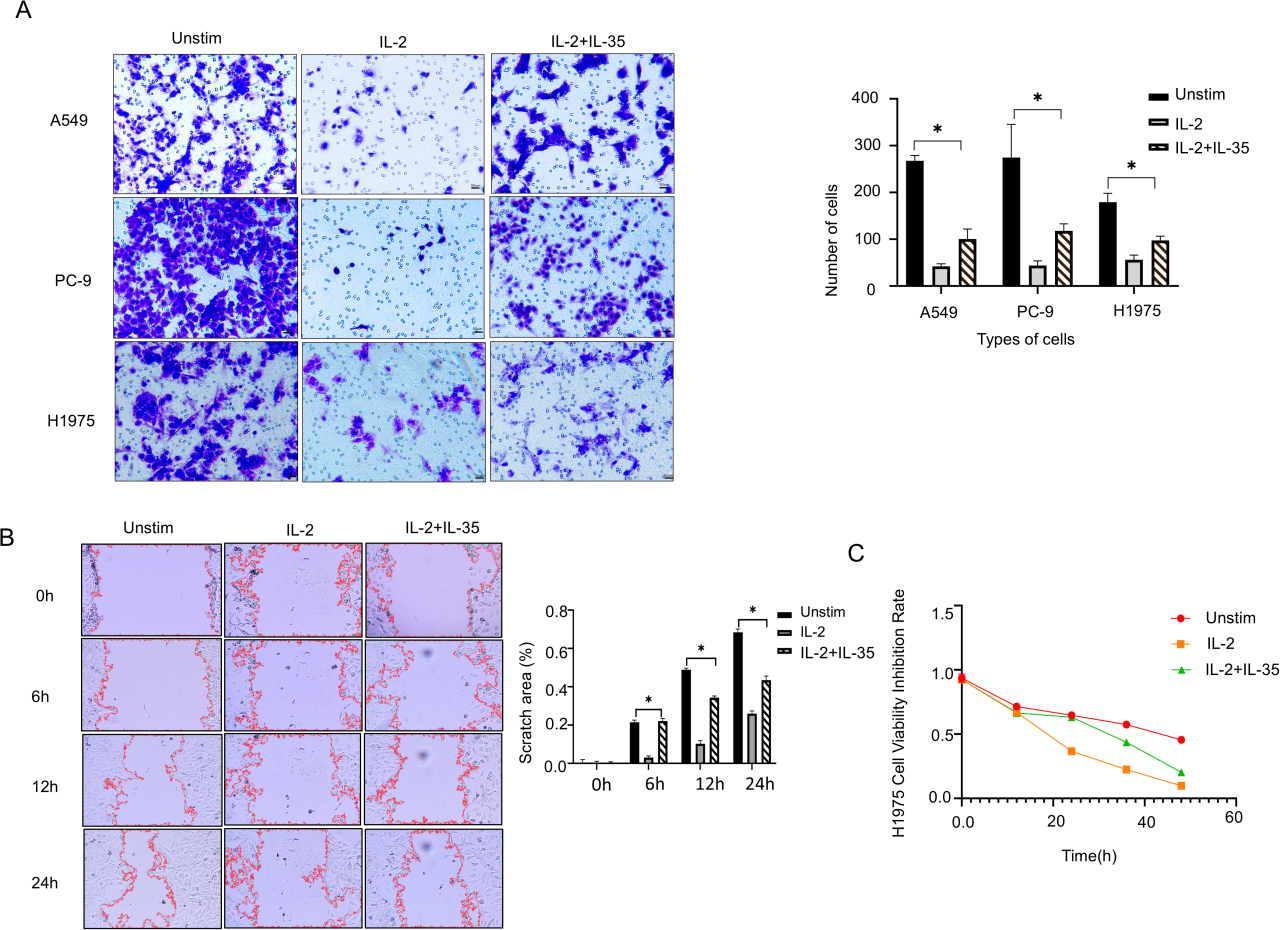
IL-35 attenuates the antitumor activity of NK cells. **(A)** Transwell invasion assays were done to assess the influence of NK cell culture supernatants from the unstimulated group, IL-2 group, and IL-2+IL-35 group on the invasive ability of A549, PC-9, and H1975 lung cancer cell lines. Representative images and quantification of invaded cells are shown. **(B)** A Wound Healing Assay Kit was selected to assess the impact of NK cell culture supernatants from the three groups on the migratory capacity of H1975 cells. Representative images were captured at 0h, 6h, 12 h and 24 h, and the wound closure area was quantified. **(C)** CCK-8 proliferation assays were conducted to make clear how NK cell culture supernatants from each group affected the proliferative ability of H1975 cells. (*) indicates statistically significant differences.

To evaluate the functional impact of IL-35 on NK cell-mediated cytotoxicity, supernatants from unstimulated, IL-2, and IL-2 + IL-35-stimulated NK cells were used to culture NSCLC cell lines A549(wild-type), PC-9(19del), and H1975(L858R-T790M). Transwell invasion assays and wound healing assays further confirmed that IL-2-stimulated NK cells suppressed the invasive and migratory capacities of all three NSCLC cell lines, and the effects were significantly attenuated by IL-35 (t-test, P < 0.05) (**Figures 4A, B**) (**Supplementary Figures 1, 2**). CCK-8 assays demonstrated that IL-2-activated NK cells significantly reduced proliferation of all three NSCLC cell lines by contrast with the unstimulated group (P< 0.05). This inhibitory effect was reversed upon IL-35 co-treatment (t-test, P < 0.05) (**Figure 4C**) (**Supplementary Figures 1, 2**).

### 3.5 IL-35 promotes NSCLC tumor growth *in vivo* and attenuates NK cell infiltration

**Figure 5:**
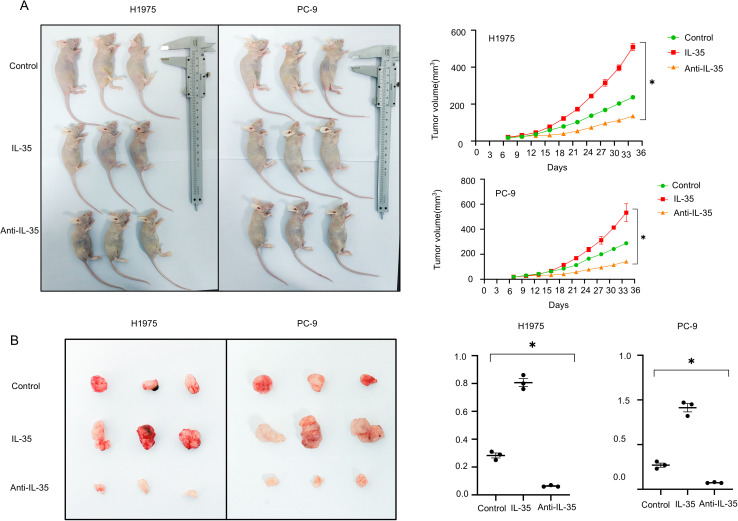
*In vivo* validation of IL-35’s effect on tumor cell growth. **(A)** An xenograft tumor model was constructed via subcutaneous injection of NSCLC 1975 and PC-9 cells in mice. The mice were randomized into the control (PBS), IL-35-treated (recombinant IL-35), and anti-IL-35 neutralizing antibody groups. Every 3days, the progression of tumor was observed, and growth curves were plotted accordingly. **(B)** Tumor weights were measured at the endpoint. Mice in the IL-35-treated group exhibited notably increased tumor mass, while those in the anti-IL-35 group showed reduced tumor weight compared to controls. (*) indicates statistically significant differences.

**Figure 6:**
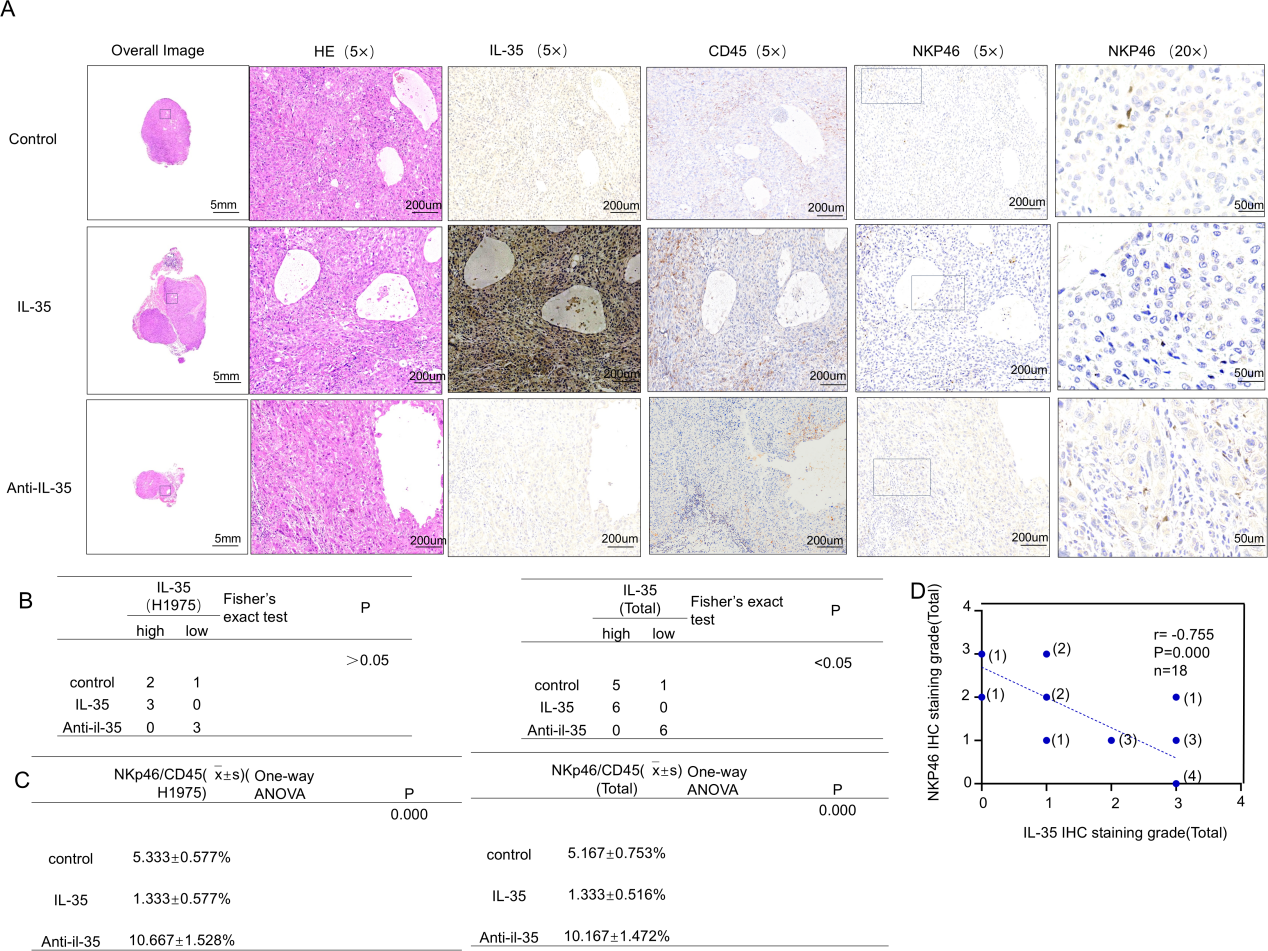
*In vivo* validation of the effect of IL-35 on NK-cell infiltration in EGFR-mutant NSCLC tumors. **(A)** Representative hematoxylin and eosin (H&E) staining and immunohistochemical **(IHC)** staining for IL-35, CD45, and NKp46 in subcutaneous H1975 xenograft tumors from the control, IL-35–treated, and IL-35–neutralizing antibody groups. **(B)** Quantification of IL-35 expression in tumor tissues from H1975 xenografts and combined Total group (H1975 and PC-9). Statistical analysis was performed using Fisher’s exact test (Monte Carlo approximation).(H1975 group: P > 0.05; Total group: P < 0.05.)**(C)** Correlation analysis of NK-cell infiltration among the control, IL-35, and IL-35–neutralizing antibody groups in H1975 and Total (H1975 + PC-9) tumors (one-way ANOVA, P = 0.000).**(D)** Correlation between IL-35 and NKp46 IHC expression in all mouse tumor samples, indicating an inverse relationship between IL-35 levels and NK-cell infiltration.

To appraise the impact of IL-35 *in vivo*, a subcutaneous xenograft was modeled on H1975 and PC-9 cells (**Supplementary Figure 4**). Mice were randomized into the control, IL-35-treated, and IL-35-neutralizing antibody-treated groups. Tumor volume and weight were substantially raised in the IL-35 group by comparison with the control (P < 0.05) (**Figures 5A, B**), whereas IL-35 neutralization caused remarkably-lower tumor progression than both IL-35-treatment and control groups (P < 0.05). Histological analysis and immunohistochemistry demonstrated that tumors from the IL-35-neutralizing antibody group exhibited lower IL-35 expression and more prominent NK cell infiltration, whereas CD45 expression showed no significant difference among groups (**Figure 6A**; **Supplementary Figure 4A**). IL-35 expression in the control, IL-35, and IL-35-neutralizing antibody groups showed no significant difference in IHC analysis of tumors derived from H1975 and PC-9 cells (**Figure 6B**; **Supplementary Figure 4B**), likely due to limited sample size; however, a significant difference was observed when the total samples from both cell lines were combined (**Figure 6B**). The proportion of NKp46^+^ cells among CD45^+^ cells was statistically significant in IHC analysis of tumors from both H1975 and PC-9 models, as well as in the combined sample set (**Figure 6C**; **Supplementary Figure 4C**). Moreover, NKp46 expression remained negatively correlated with IL-35 expression (**Figure 6D**; **Supplementary Figures 4D, E**).

## 4 Discussion

EGFR-mutated NSCLC is characterized by a well-defined tumor immune microenvironment (TIME) that is generally less immunogenic and more immunosuppressive compared to EGFR wild-type counterparts (12). It has been revealed by existing references that EGFR mutations can facilitate the recruitment and expansion of tumor-associated macrophages (TAMs), myeloid-derived suppressor cells (MDSCs), and regulatory T cells (Tregs) among other immunosuppressive cell types (13). Such cells secrete a variety of cytokines that suppress immune responses including TGF-β, IL-10, and VEGF, which collectively contribute to immune evasion and tumor progression. In our study, we identified IL-35 as a novel and significant contributor to the immunosuppressive milieu in EGFR-mutant NSCLC. We found that IL-35 is predominantly secreted by immunosuppressive cell subsets infiltrating the tumor tissue. Notably, EGFR mutation status correlated positively with increased infiltration of these immunosuppressive cells and, consequently, with elevated IL-35 expression in tumor tissues. These findings suggest that EGFR mutations influence tumor cell behavior in a direct manner other than indirectly shaping TIME by promoting the accumulation of immunosuppressive cells and the secretion of IL-35. Our data distinctly indicate that IL-35 expression is substantially up-regulated in EGFR-mutant NSCLC rather than EGFR wild-type tumors, highlighting IL-35 as a potential mediator of EGFR mutation-associated immunosuppression. Moreover, our findings demonstrated that in both the overall population and the EGFR-mutant subgroup, patients with high IL-35 expression exhibited significantly different survival outcomes compared to those with low IL-35 expression (P < 0.05), indicating that IL-35 expression levels may influence patient prognosis.

IL-35 is a well-characterized immunosuppressive cytokine known to inhibit the function of various immune cells, particularly T lymphocytes (14, 15). However, its role in regulating NK cell function remains largely unexplored. The present study offers novel insights into the immunosuppressive influence of IL-35 on NK cells in the context of NSCLC. A remarkably negative mutuality was discovered between the level of IL-35 and the presence of NK cells, as indicated by the NK cell-specific marker NKp46 (16–19), in tumor tissues. NKp46 is a specific lineage marker for NK cells, while CD45 labels all leukocytes (20). Calculating the NKp46^+^/CD45^+^ ratio normalizes NK cell infiltration to total immune cells, allowing consistent comparison of NK representation across lung cancer tissues despite variable leukocyte densities. This study shows that in EGFR-mutant NSCLC tissues, elevated IL-35 expression is accompanied by a decreased NKp46/CD45 ratio. To further investigate this relationship, isolation of peripheral blood mononuclear cells (PBMCs) and expansion of NK cells were performed *in vitro*. CCK8 assays revealed that IL-35 significantly inhibited NK cell proliferation. Through flow cytometry analysis, it was suggested that IL-35 reduced the proportion of NK cells and downregulated the expression of the activating receptor NKG2D (21, 22). Additionally, ELISA assays showed that IL-35 treatment markedly decreased the secretion of IFN-γ, perforin, granzyme B and some other key NK cell effector molecules (7). These findings collectively indicate that IL-35 exerts potent immunosuppressive effects on NK cells by impairing their proliferation, activation, and effector functions.

IL-35-Impaired NK Cell Function Promotes Tumor Progression. NK cells are instrumental in immunity against tumors by directly killing tumor cells and secreting cytokines such as IFN-γ, perforin, and granzyme B. To estimate the functional consequences of IL-35-mediated NK cell suppression, we performed co-culture experiments using NK cell supernatants and NSCLC cell lines (PC-9, A549 and H1975). NK cells were cultured under three conditions: unstimulated, IL-2-stimulated, and IL-2 combined with IL-35. The supernatants from these cultures were then used to treat NSCLC cells. It was demonstrated by our findings that supernatants from IL-35-treated NK cells significantly attenuated the ability of NK cells to suppress tumor cell proliferation, invasion, and migration. This suggests that IL-35 suppresses NK cell function and indirectly promotes tumor progression by reducing the effectiveness of NK cells in inhibiting cancerous tumors. These observations underline the key role of IL-35 in mediating immune evasion and tumor progression in EGFR-mutant NSCLC.

Given the established role of EGFR mutations in promoting an immunosuppressive TIME (23, 24)and the limited effectiveness of immune checkpoint inhibitors (ICIs) covering anti-PD-1/PD-L1 therapies in EGFR-mutant NSCLC (25, 26), there is a pressing need to develop innovative treatment targets. Our study indicates IL-35 could act as a promising immunotherapy target in this context. We established a subcutaneous tumor model using the H1975 and PC-9 NSCLC cell lines and treated mice with either recombinant IL-35 (27), an IL-35-neutralizing antibody (28), or vehicle control. When compared to controls, mice having been administered an IL-35-neutralizing antibody exhibited much less tumor growth. Immunohistochemical analysis revealed decreased IL-35 expression and increased NK cell infiltration in tumors from the antibody-treated group. These results indicate that neutralizing IL-35 can bring back NK cell function and strengthen immunity against tumors, thereby inhibiting tumor progression. Although our findings identify IL-35 as a key immunosuppressive cytokine that impairs NK cell function in the EGFR-mutant non–small cell lung cancer (NSCLC) microenvironment, several limitations and potential confounding factors should be acknowledged to ensure proper interpretation of the results. First, the *in vitro* experimental conditions employed in this study may not fully recapitulate the complexity of the *in vivo* tumor microenvironment. The concentrations and exposure durations of IL-35 applied to cultured NK cells may differ from physiological levels within tumor tissues. Second, indirect effects mediated by other immunoregulatory factors—including TGF-β, IL-10, and adenosine—cannot be excluded. These secondary effects may further suppress NK cell activity, complicating the distinction between direct and indirect mechanisms.

## 5 Conclusion

In conclusion, our study demonstrates that IL-35, secreted by immunosuppressive cells recruited in response to EGFR mutations, contributes to suppressing NK cell function and promoting tumor progression in NSCLC. For patients with EGFR-mutant NSCLC, targeting IL-35 may serve as a new and efficient therapeutic approach to overcome immunosuppression and enhance clinical outcomes.

## Data Availability

The datasets presented in this article are not readily available. The data that support the findings of this study are available from the corresponding author upon reasonable request. Requests to access the datasets should be directed to zhangyan20250819@163.com.
