# Supplementary material for: Suppression of NK cell-mediated immunosurveillance by IL-35 drives tumor progression in EGFR-mutant non-small cell lung cancer
Source: Front Oncol. 2025 Nov 20;15:1692801. doi: 10.3389/fonc.2025.1692801 (PMC12676456; doi:10.3389/fonc.2025.1692801)
Supplement: Supplementary file 1 [file DataSheet1.docx]

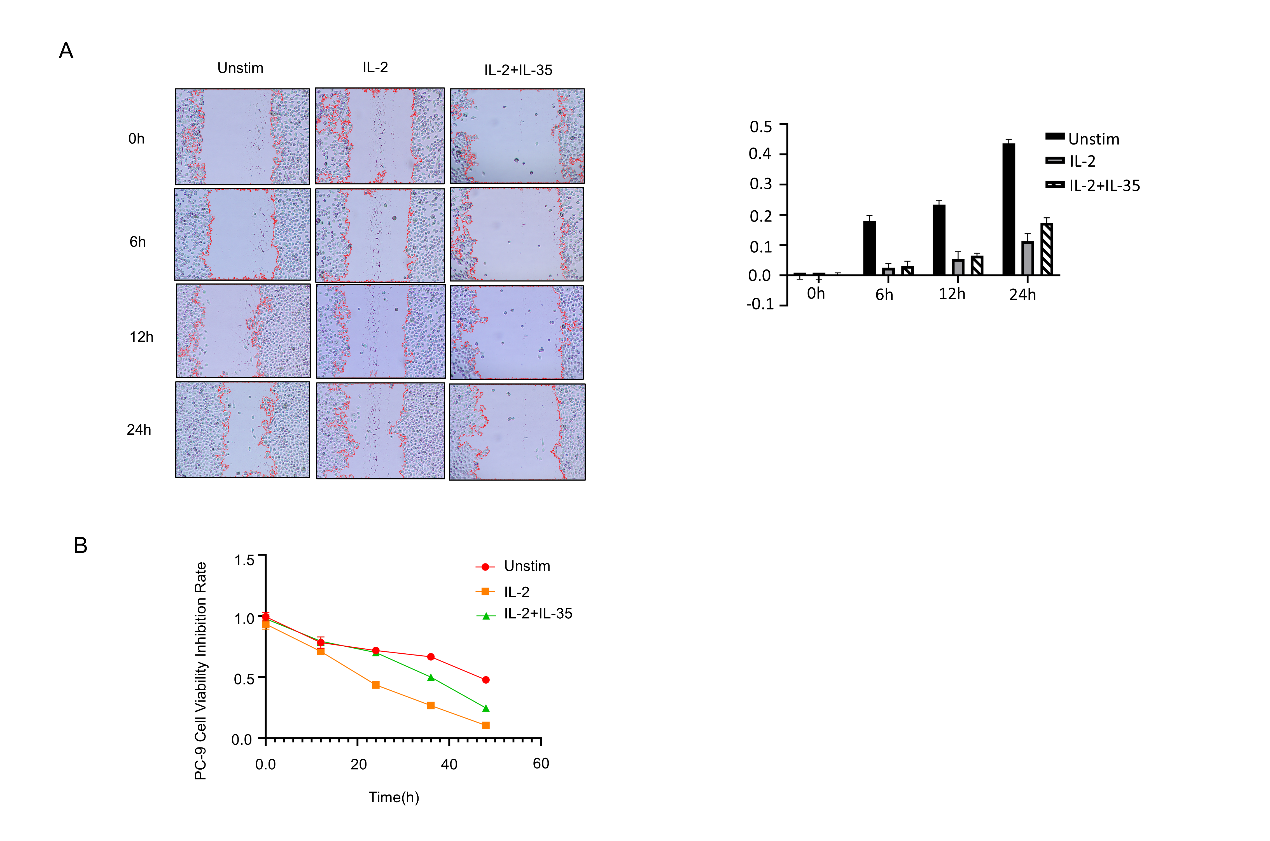


**Supplementary Figure 1 | IL-35 attenuates the antitumor activity of NK cells.**
**(A)** A Wound Healing Assay Kit was selected to assess the impact of NK cell culture supernatants from the three groups on the migratory capacity of PC-9 cells. Representative images were captured at 0h, 6h, 12 h and 24 h, and the wound closure area was quantified. **(B)** CCK-8 proliferation assays were conducted to make clear how NK cell culture supernatants from each group affected the proliferative ability of PC-9 cells..


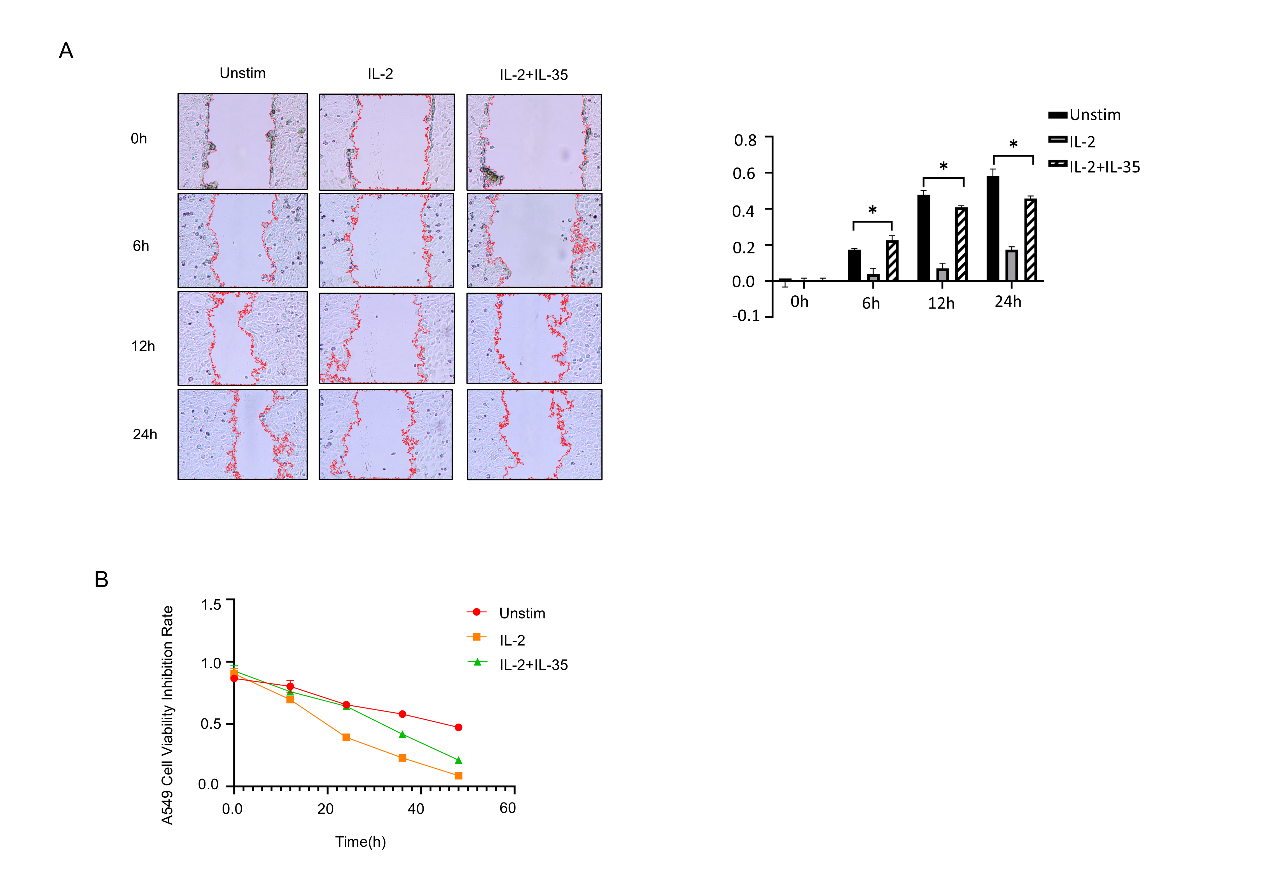


**Supplementary Figure 2 | IL-35 attenuates the antitumor activity of NK cells.**

**(A)** A Wound Healing Assay Kit was selected to assess the impact of NK cell culture supernatants from the three groups on the migratory capacity of A549 cells. Representative images were captured at 0h, 6h, 12 h and 24 h, and the wound closure area was quantified. **(B)** CCK-8 proliferation assays were conducted to make clear how NK cell culture supernatants from each group affected the proliferative ability of A549 cells.


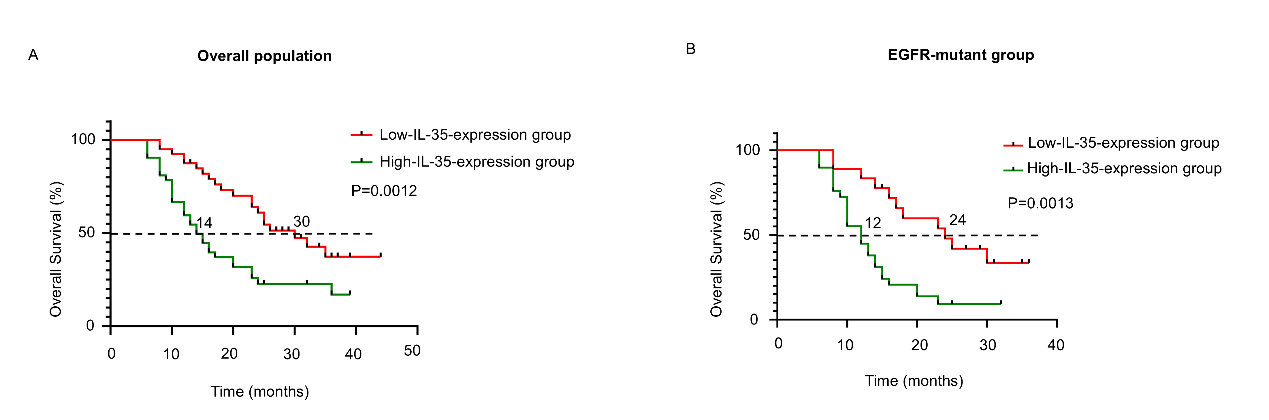


**Supplementary Figure 3. Correlation between IL-35 expression and overall survival in patients with non–small cell lung cancer (NSCLC).**

(A) Kaplan–Meier survival analysis of all NSCLC patients showing that those with high IL-35 expression had significantly shorter overall survival than those with low IL-35 expression (P = 0.0012).

(B) Kaplan–Meier survival analysis of EGFR-mutant NSCLC patients demonstrating that high IL-35 expression was associated with significantly poorer overall survival compared with low IL-35 expression (P = 0.0013).


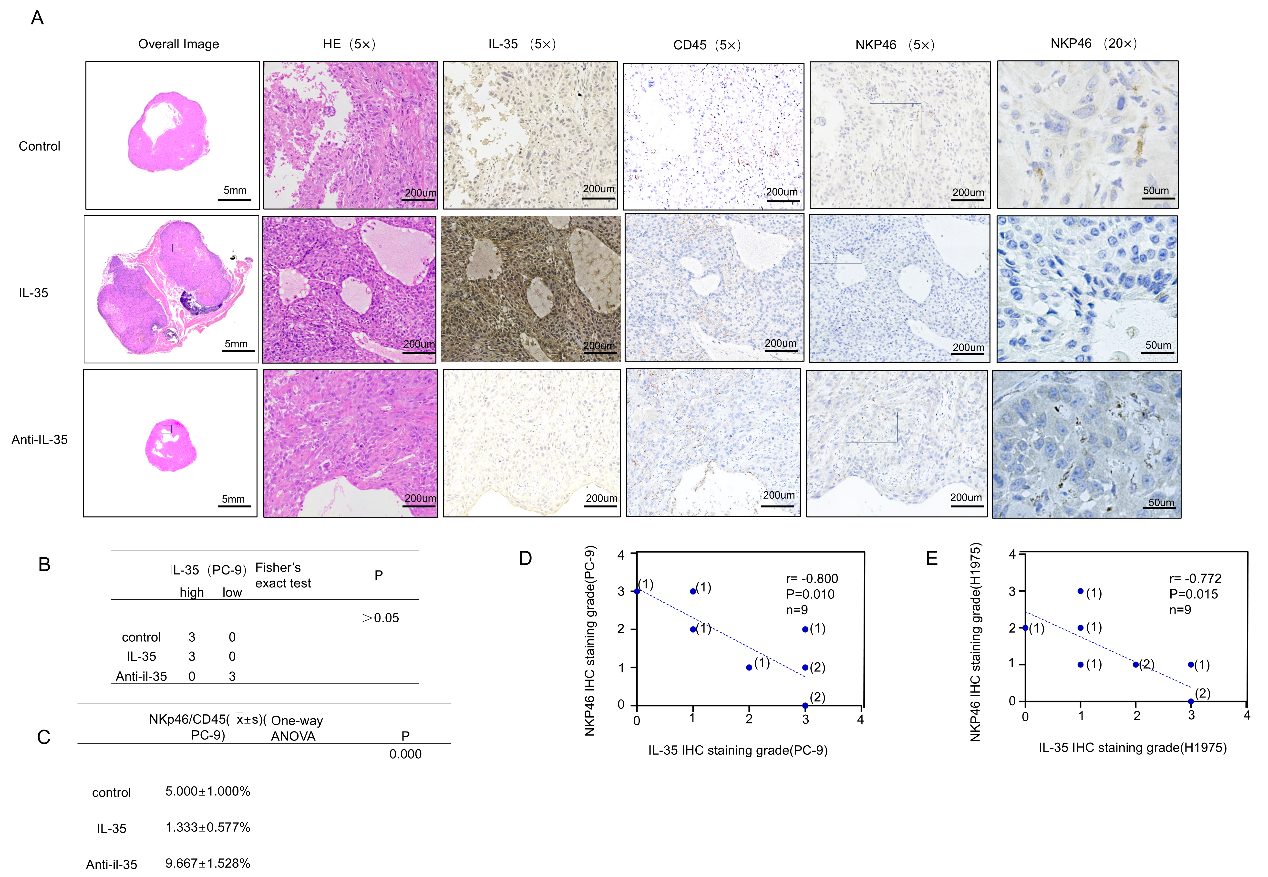


**Supplementary Figure 4. In vivo validation of the effect of IL-35 on NK-cell infiltration in tumor tissues.**

(A) Representative images of hematoxylin–eosin (H&E) staining and immunohistochemical staining for IL-35, CD45, and NKP46 in subcutaneous xenograft tumors derived from PC-9 cells in the control, IL-35-treated, and anti–IL-35-neutralizing antibody groups.(B) Comparison of IL-35 expression levels among the three groups in the PC-9 xenograft model (Fisher’s exact test, Monte Carlo approximation; P > 0.05).(C) Quantitative analysis of NK-cell infiltration among the control, IL-35, and anti–IL-35 groups in PC-9 xenografts (one-way ANOVA; P = 0.000).(D) Correlation between IL-35 expression and NKP46⁺ NK-cell infiltration in PC-9 tumor tissues (r = –0.800, P = 0.010).(E) Correlation between IL-35 expression and NKP46⁺ NK-cell infiltration in H1975 tumor tissues (r = –0.772, P = 0.015).
